# Influence of competition and intraguild predation between two candidate biocontrol parasitoids on their potential impact against Harrisia cactus mealybug, *Hypogeococcus* sp. (Hemiptera: Pseudococcidae)

**DOI:** 10.1038/s41598-021-92565-6

**Published:** 2021-06-28

**Authors:** María B. Aguirre, Octavio A. Bruzzone, Serguei V. Triapitsyn, Hilda Diaz-Soltero, Stephen D. Hight, Guillermo A. Logarzo

**Affiliations:** 1 Fundación para el Estudio de Especies Invasivas (FuEDEI), Bolívar 1559 (1686), Hurlingham, Buenos Aires Argentina; 2 Consejo Nacional de Investigaciones Científicas y Técnicas, Ciudad Autónoma de Buenos Aires, Buenos Aires, Argentina; 3 Instituto Nacional de Tecnología Agropecuaria (INTA), Estación Experimental Agropecuaria Bariloche, San Carlos de Bariloche, Río Negro Argentina; 4 Department of Entomology, University of California, Riverside, CA 92521 USA; 5 Animal and Plant Health Inspection Service USDA, San Juan, Puerto Rico; 6 USDA-ARS-CMAVE at Center for Biological Control, Florida A&M University, Tallahassee, FL 32308 USA

**Keywords:** Behavioural ecology, Animal behaviour, Entomology

## Abstract

When two or more parasitoid species, particularly candidates for biocontrol, share the same target in the same temporal window, a complex of behaviors can occur among them. We studied the type of interactions (competition and intraguild predation) that existed between the nymphal parasitoids *Anagyrus cachamai* and *A. lapachosus* (Hymenoptera: Encyrtidae), two candidate neoclassical biocontrol agents against the Puerto Rican cactus pest mealybug, *Hypogeococcus* sp. (Hemiptera: Pseudococcidae). The surrogate native congener host in Argentina, the cactus mealybug *Hypogeococcus* sp., was studied to predict which species should be released; in the case that both should be released, in which order, and their potential impact on host suppression. In the laboratory we conducted experiments where different densities of the host mealybug were exposed to naive females of *A. cachamai* and *A. lapachosus* sequentially in both directions. Experiments were analyzed by combining a series of competitive behavioral and functional response models. A fully Bayesian approach was used to select the best explaining models and calculate their parameters. Intraguild predation existed between *A. cachamai*, the species that had the greatest ability to exploit the resource, and *A. lapachosus*, the strongest species in the interference competition. The role that intraguild predation played in suppression of *Hypogeococcus* sp. indicated that a multiple release strategy for the two biocontrol agents would produce better control than a single release; as for the release order, *A. lapachosus* should be released first.

## Introduction

Intraguild predation is a combination of exploitative competition and predation among potential competitors that use the same host or prey^1,2^, either a uni- or bidirectional interaction. When unidirectional, one of the interacting species is called the intraguild predator (a natural enemy that attacks another natural enemy species) and the other is the intraguild prey (the natural enemy attacked). In bidirectional cases each species fulfils both roles^3^. Intraguild predation may occur among only predators, only parasitoids, or different combinations of parasitoids and predators. When this interaction occurs between parasitoids and predators, it is typically asymmetrical, the parasitoid is always the subordinate species, a prey. This can have particular relevance in suppression of herbivorous insects since it can impact the population dynamics of both the natural enemies and the pest^4^. If one species simply kills the other without feeding on it, the interaction is qualified as interspecific killing, an extreme form of interference competition^5^. To ascertain the potential occurrence of intraguild predation in parasitoid-parasitoid interactions, the experimental design must quantify immediate fitness benefits of the species involved in the interaction. Predation of the intraguild prey should provide direct nutritional and energetic gains for the intraguild predator, which are reflected in increased growth, reproduction, or survival^2^.

The study of intraguild predation has become relevant in biological control programs because it can have negative consequences on pest mortality^6,7^. Although this problem could be avoided by the introduction of a single biological control agent, several reasons may promote the release of more than one agent, such as lack of efficacy of the biocontrol agent, low establishment rate^8^, or simply natural enemies present in the release area that negatively interact with the one agent^9^.

There is currently no consensus on the role of intraguild predation regarding the success of biological control programs^10,11^. The first theoretical models developed on intraguild predation analyzed the changes that occurred in the equilibrium of the populations of the intraguild predator, the intraguild prey, and in the resource shared by both (i.e., the pest), as a function of the environmental productivity^12–17^. According to these models, if the intraguild predator is a weaker resource competitor (inferior natural enemy) than the intraguild prey, both can coexist, or even exclude the intraguild prey. In both cases, the equilibrium density of the pest will increase as a result of the reduction of the intraguild prey, the natural enemy that has the greatest ability to exploit the resource (the pest). When the intraguild predator is a superior natural enemy of the exploitative competition, the intraguild predator will exclude the intraguild prey. In this case, there is no negative effect of the intraguild predation on the resulting biological control, and there is no benefit in releasing the intraguild prey to control the pest^4^. Janssen et al.^18^ when reviewing the theory of intraguild predation and its consequences on biological control, found that the resulting predictions from the above theoretical models were not confirmed in many of the empirical studies they analyzed. The authors considered, as possible causes of this discrepancy between theory and results, that the theoretical models do not take into account the effect of the antipredatory behavior of the intraguild prey and the pest, the complexity of the food chain, and the temporal and spatial scale analyzed. Mechanistic models of population, community and ecosystem dynamics require the mathematical description of trophic interactions in the form of functional response equations, which allows describing the number of hosts/preys attacked by a parasitoid/predator in relation to host/prey abundance. In fact, intraguild predation is strongly related to functional response. If the shared resource is abundant, the overlap between the intraguild predator and the intraguild prey would be rare, but if the resource is scarce, the interaction is inevitable. Although some studies integrated the inter- and intraspecific competition into the functional response models, there are no models that consider intraguild predation, or multiple interactions^19,20^. Functional response studies should include behavioral interactions among multiple consumer species or types of resources to improve their predictive power. So, just as population biology has gained enormously from the incorporation of individual processes in population models^21^, mechanistic understanding in community ecology can be increased by incorporating behavioral studies at the interspecific level into community models via multi specific functional response equations^20^. In biological control programs, this type of analysis could increase knowledge of interactions of the candidate species with each other and effects of their interactions on control of the pest. This information is of the utmost importance when there are several candidate species being considered in a biological control program since there is a wide range of potential interactions that could influence their performance^22^.

Parasitoid competition can be extrinsic (adult-adult) or intrinsic (adult-larva or larva-larva)^23^; it is usually studied through laboratory experiments, where hosts are exposed to parasitic wasps at different sequences and combinations. One of the most common difficulties of these kinds of experiments is to elucidate what happens inside the host^24^ and the effect of host availability on the ability of the wasps to locate the hosts^25^. To solve this problem, Bruzzone et al.^26^ proposed a novel approach that integrates competitive behavioral and functional response models. The models developed allow the description of the competition process of endoparasitoids both on interference (which species is a better interference competitor, if the competitor has advantages by arriving first; and when arriving second, whether the parasitoid avoids, accepts, or prefers the already parasitized hosts), and exploitation (if there are differences in terms of functional response).

The aim of this study was to analyze the type of interactions (competition and intraguild predation) that existed between two parasitoid species, both promising biological control candidates, and the effect of these interactions on mortality of the shared host. To reach the objective, the methodological approach proposed by Bruzzone et al.^26^ was used. We particularly analyzed the intrinsic interactions (adult-larva and larva-larva) of *Anagyrus cachamai* Triapitsyn, Logarzo & Aguirre and *A. lapachosus* Triapitsyn, Aguirre & Logarzo (Hymenoptera: Encyrtidae), candidate agents for the neoclassical biological control of the Harrisia cactus mealybug *Hypogeococcus* sp. (Hemiptera: Pseudococcidae), a Brazilian native turned invasive pest of the native cacti in Puerto Rico^27–29^. With the information obtained, we predicted if both parasitoid species should be released and the level of their potential impact on the target pest.

## Methods

The studies were conducted at Fundación para el Estudio de Especies Invasivas (FuEDEI) facilities, located in Hurlingham, Buenos Aires, Argentina, between April 2015 and February 2017. All the experiments and insect rearing were carried out under laboratory-controlled conditions (25 ± 1 °C, 16:8 L:D, 60–80% RH), except stated otherwise.

### Parasitoid rearing

Laboratory studies were carried out with colonies of the parasitoid species *A. cachamai* and *A. lapachosus* reared at FuEDEI facilities since 2014 with the methodology described in Aguirre et al.^28^. Both parasitoids were reared separately on first instar nymphs of *Hypogeococcus* sp. “*Cactaceae host-clade*”^30^, a congener but a different species from the pest *Hypogeococcus* sp. in Puerto Rico. Mealybug colonies were reared on clean potted plants of *Cleistocactus baumannii* (Lem.) Lem. (Cactaceae). All observations were conducted under a dissecting microscope at 40×.

Colonies of these two encyrtid species were reared in separate chambers. Four mated females of the same parasitoid species were placed in a plastic cage (2 L) with a hole in the lid (6 cm diameter) covered with polyester gauze for ventilation. The cage contained an excised piece of *C. baumannii* (20–25 cm long) infested with about 100 first instar nymphs of *Hypogeococcus* sp. “*Cactaceae host-clade*”, obtained from separate mealybug colonies. After 72 h, the four female wasps were removed from the cage, and the nymphs exposed to the parasitoids were monitored every 3 days. Once the first pupa was detected, monitoring was conducted daily, and all parasitoid pupae found were removed and transferred to a Petri dish (1.5 cm high × 5.5 cm diameter) covered with plastic food wrap to keep wasps from escaping after emergence. In this way, it was possible to control the wasps’ age, mating, and feeding conditions. The emerged wasps were transferred to a new Petri dish of equal dimensions, with a squashed drop of honey on the bottom, and covered with clear plastic food wrap, either for rearing or experimental purposes. The age of the female parasitoids for the experiments was 24–48 h old; they were fed, mated, and had no previous oviposition experience. From now on, when we mention nymphs of *Hypogeococcus* sp. exposed to female parasitoids, we refer to first instar nymphs of *Hypogeococcus* sp. “*Cactaceae host-clade*” on 20–25 cm long pieces of *C. baumannii*.

### Interspecific parasitoid interaction experiments

These studies were performed by integrating functional response and competition experiments. Consequently, different *Hypogeococcus* sp. nymph densities were exposed to *A. cachamai* and *A. lapachosus* sequentially in both ways. As a result, two functional response curves were obtained for the interaction, one where the nymphs were first exposed to females of *A. cachamai* and then the same nymphs were exposed to females of *A. lapachosus*. The second functional response curve was the reciprocal experiment where nymphs were first exposed to *A. lapachosus.* At the same time, the potential impact of each of the two studied parasitoids on the target pest was estimated with a standard functional response trial conducted with each parasitoid species, ergo the number of hosts attacked as a function of hosts offered to each parasitoid species without interaction was estimated. The functional response curves in the absence of interaction were used as a baseline to the treatments of consecutive exposures, and they also served as a null model in which the absence of interference between the hosts was postulated.

To estimate the functional response curves of the interaction between *A. cachamai* and *A. lapachosus*, *Hypogeococcus* sp. nymphs were exposed at a constant density to a female of one parasitoid species (*A. cachamai* or *A. lapachosus*) for 24 h, at the end of which, the female was removed and the nymphs were exposed to a female of the second, different species for another 24 h. The reciprocal exposure for the two wasp species was also performed. Both exposure combinations were conducted at 10 different nymph densities: 10, 20, 30, 40, 50, 60, 70, 80, 90 and 110, with a maximum error of 10% in the number of nymphs per density offered. The densities were determined based on the results of a pilot test, where densities of 80 and 110 nymphs produced a plateau in the curve of the number of nymphs attacked as a function of the density offered. The nymphs exposed to both *A. cachamai* and *A. lapachosus* females were monitored every 3 days until all non-parasitized nymphs completed their development and wasps emerged from the parasitized nymphs. For each density tested, the number and species of parasitoids emerged and the number of non-parasitized nymphs was recorded. Functional response of each species in the absence of interaction (baseline) was estimated with an experimental design similar to that explained above, with the difference that nymphs were not exposed to a second female of the alternative species. The densities used for the construction of the functional response curves were 10, 20, 40, 60, 80 and 110 nymphs (with a maximum error of 10% in the number of nymphs per density offered), and each density was replicated five times. The experiments were conducted in vented plastic cages similar to the one described for the parasitoid rearing.

### Data analyses

The outcome of the interspecific parasitoid interaction experiments was analyzed through a Bayesian process of model selection. This analysis procedure started with a simple model of type one functional response, adding terms until an optimal model was obtained resulting in a balance between explanatory power and complexity. The result was the identification of the competitive relationship between parasitoids (Bruzzone et al.^26^; see appendix section for details on the models used (Supplementary).

The main reason for using this approach was the lack of complete control of the number of hosts offered in each trial, and the inability to directly observe the interaction between parasitoid larvae since both parasitoid species are endoparasitoids and the interaction occurred inside very small individual nymphs (0.35 ± 0.06 mm). To address these drawbacks of the experimental setup, we opted to model the number of expected emerging parasitoids using a functional response model to estimate the expected number of hosts attacked by each parasitoid species. Once we had an estimate of the number of hosts attacked, it was possible to estimate the overlap degree of the hosts attacked by both parasitoid species, estimating in turn the competition process between hosts by comparing the number of parasitoids emerged with those expected through different competition models.

First, we estimated the number of nymphs attacked as a function of the nymphs offered; consequently, an estimate of the parasitoid functional response was needed. A host selection model using a set theory was necessary to identify how the female parasitoid chose its hosts, and its output was applied to a well-known model of competition, in this case, the Thurstone/Bradley Terry Model^31,32^.

The base model of the interspecific parasitoid interaction experiments between the first species to arrive (species 1), and the second to arrive (species 2) was as follows:

1 $${E}_{1/2}\left(p\right)={R}_{10}(p)+{R}_{12}(p)\left(1-{w}_{12}\right)$$

where ${R}_{12}(p)={R}_{1}\left(p\right)\cap {R}_{2}\left(p\right)$, where *E*_1/2_ is the expected number of species 1 emerged, given that species 2 also attacks the same host, and *p* is the number of hosts available. *R*_1_(*p*) is the functional response of species 1; *R*_2_(*p*) is the functional response of species 2; and *w*_12_ is the proportion of times in which species 1 won in the competition against species 2. The term *R*_10_(*p*) represents the hosts attacked by species 1 that are not attacked by species 2, and *R*_12_*(p)* are the hosts attacked by species 1 that are later attacked by species 2, so *R*_1_(*p*) = *R*_10_(*p*) + *R*_12_(*p*). The whole term ${R}_{1}\left(p\right)\cap {R}_{2}(p)$ is the expected superposition of the functional responses of species 1 and 2.

The functional response model gives the proportion of hosts attacked by each parasitoid, which allowed us to describe the exploitation competition. The competition model provides the proportions of each species of parasitoid that emerges from the hosts attacked by both, which allowed us to analyze whether interference competition or intraguild predation existed. The interference competitive behavior was divided into three dimensions: competitive rating, the effect in the order of arrival to the host in the parasitoid’s competitive strength, and a parasitoid superposition model (an estimator of the degree of utilization/rejection of already parasitized hosts). We also modeled whether host mortality was constant and independent of the number of parasitoids that attacked a host, and if parasitoids were able to differentiate between suitable and unsuitable hosts (Supplementary, Equation (8)). Finally, we performed a stepwise selection of the proposed models in order to find which one had the best balance between explanation of the data (in terms of the likelihood function) and complexity (in terms of number of parameters).

The selection of models and the estimation of parameter distribution were conducted in a Bayesian framework. We performed the model selection procedure using the algorithm Reversible Jump Markov-Chain Monte Carlo, in which the routine automatically “jumped” from one model to another and then selected the best model balancing information and fitting. To achieve this, in each jump, for each additional parameter, the log-likelihood function was penalized with a value of minus two. The first 40,000 iterations of the reversible procedure were discarded as a burn-in model selection, and the last 20,000 were used to calculate the weight of each model in the model averaging procedure. Also, 10,000 iterations of Markov Chain Monte Carlo were performed for each iteration of the Reversible Jump algorithm^33^, resulting in a total of 60,000,000 iterations. The last 20,000,000 iterations were used to calculate a posteriori distribution of the parameters. Expected vs. observed values were compared using a binomial likelihood function for the number of the parasitoids emerged in relation to the total hosts offered. The a priori distribution of the parameters of the functional response curves were a non-informative uniform distribution between 0 and 1, the same for the multiparasitism index. On the other hand, for the competition parameters, the a priori distribution was a normal distribution with mean 0 and deviance 10 for all the parameters since we did not have a priori information of the variables distribution. As in the studies of competitive behavior, the strength of each species is an “interval scale” and therefore does not have an origin ordinate^34^. The variable of the species *A. cachamai* was arbitrarily fixed as 0 and used as a reference of the competitive strength of *A. lapachosus* (the ordinate of origin was the *A. cachamai* strength, and the interval unit was the standard deviation of that species’ strength). All analyses were performed using a PyMC library for Bayesian estimation^35^ in the Python programming language.

## Results

### Interspecific parasitoid interaction experiments

The results of the laboratory experiments are shown in Table 1. In the absence of interaction, both *A. lapachosus* and *A. cachamai* females parasitized around 24% of *Hypogeococcus* sp. exposed nymphs. In the presence of interaction, *A. lapachosus* attacked 20.1% of the nymphs offered when *A. cachamai* females were released first. In the reciprocal experiment, when *A. lapachosus* was released first, 23.89% of host nymphs were parasitized by *A. lapachosus*. Parasitism by *A. cachamai* revealed an attack of 18.94% of the nymphs offered when *A. lapachosus* females were released first and an attack of 11.51% of the offered nymphs when *A. cachamai* was released first. The percentage of parasitism produced by a single species was lower than when two species were sequentially introduced in the arena, regardless of the order of release (Table 1).

**Table 1:** Parasitism of *Hypogeococcus* sp. nymphs by two parasitoids, *Anagyrus cachamai* and *A. lapachosus*, when released alone or sequentially.

Wasp species released		No. nymphs exposed	No. parasitoids emerged	Parasitoids emerged (%)		
First	Second			First	Second	Total
*Anagyrus cachamai*	–	1946	466	23.95	–	23.95
*Anagyrus lapachosus*	–	1981	483	24.38	–	24.38
*Anagyrus cachamai*	*Anagyrus lapachosus*	582	184	11.51	20.10	31.61
*Anagyrus lapachosus*	*Anagyrus cachamai*	586	251	23.89	18.94	42.83

### Data analyses

From the 112 proposed models to analyze the results of the laboratory experiments, eight models were selected via the reversible jump procedure (Table 2). These integrated models comprised between 14 and 16 biological parameters that explained the behavior of wasps when they shared the same host; first instar nymphs of *Hypogeococcus* sp. All the models selected indicated that *A. cachamai* and *A. lapachosus* competed with each other.

**Table 2:** Proposed models analyzed to identify the kind of interactions (competition and intraguild predation) between two species of *Hypogeococcus* sp. parasitoids, *Anagyrus cachamai* and *A. lapachosus*.

Models of functional response	Models with increase in host mortality	Models of competition							
		Absent	Present, without parasitoids superposition	Present, without parasitoids superposition and competitive advantage by the order of arrival to the host	Present, with parasitoids superposition and competitive advantage by the order of arrival to the host	Absent	Present, without parasitoids superposition	Present, without parasitoids superposition and competitive advantage by the order of arrival to the host	Present, with parasitoids superposition and competitive advantage by the order of arrival to the host
		Models without rejection of non-suitable hosts				Models with rejection of non-suitable hosts			
FRI, NHD	Absent	2	4	7	10	3	5	8	11
FRII, NHD		4	6	9	12	5	7	10	13
FRII, HD		4	6	9	12	5	7	10	13
FRIII GP, NHD		6	8	11	14	7	9	12	15
FRIII GP, HD		6	8	11	14	7	9	12	15
FRIII G, NHD		6	8	11	14 **(15.8%)**	7	9	12	15 **(21.3%)**
FRIII G, HD		6	8	11	14 **(10.2%)**	7	9	12	15 **(11.8%)**
FRI, NHD	Present	3	5	8	11	4	6	9	12
FRII, NHD		5	7	10	13	6	8	11	14
FRII, HD		5	7	10	13	6	8	11	14
FRIII GP, NHD		7	9	12	15	8	10	13	16
FRIII GP, HD		7	9	12	15	8	10	13	16
FRIII G, NHD		7	9	12	15 **(21.4%)**	8	10	13	16 **(8.2%)**
FRIII G, HD		7	9	12	15 **(8.2%)**	8	10	13	16 **(3.1%)**

Combinations of models were tested resulting in 112 different models (see Supplementary for model development). Values are the number of parameters for each model combination, and in bold and parenthesis the weight of the eight selected models for model averaging after 20,000 reversible jump iterations.

NHD identified models without host depletion and HD with host depletion. GP: order two polynomial generalized type III functional response; G: generalized type III functional response.

**Figure 1:**
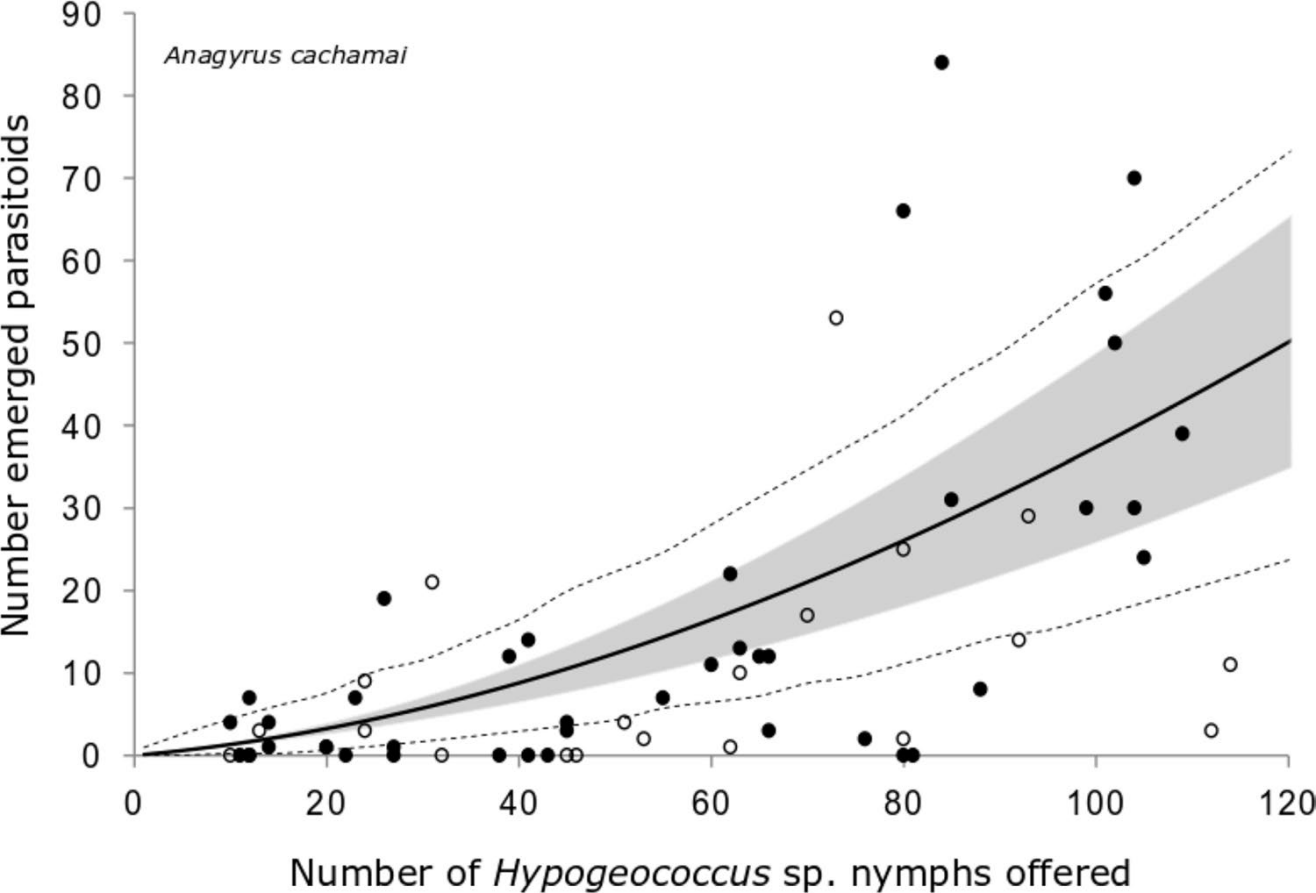
Observed functional response of the parasitoid *Anagyrus cachamai* attacking *Hypogeococcus* sp. nymphs*.* Solid line indicates the mean estimation of functional response, grey area indicates its credibility interval, and dashed line indicates the a posteriori credibility interval for individual measurements. White circles are the observed number of emerged parasitoids in interaction experiments, while dark circles are the number of parasitoids emerged in experiments without interaction.

**Figure 2:**
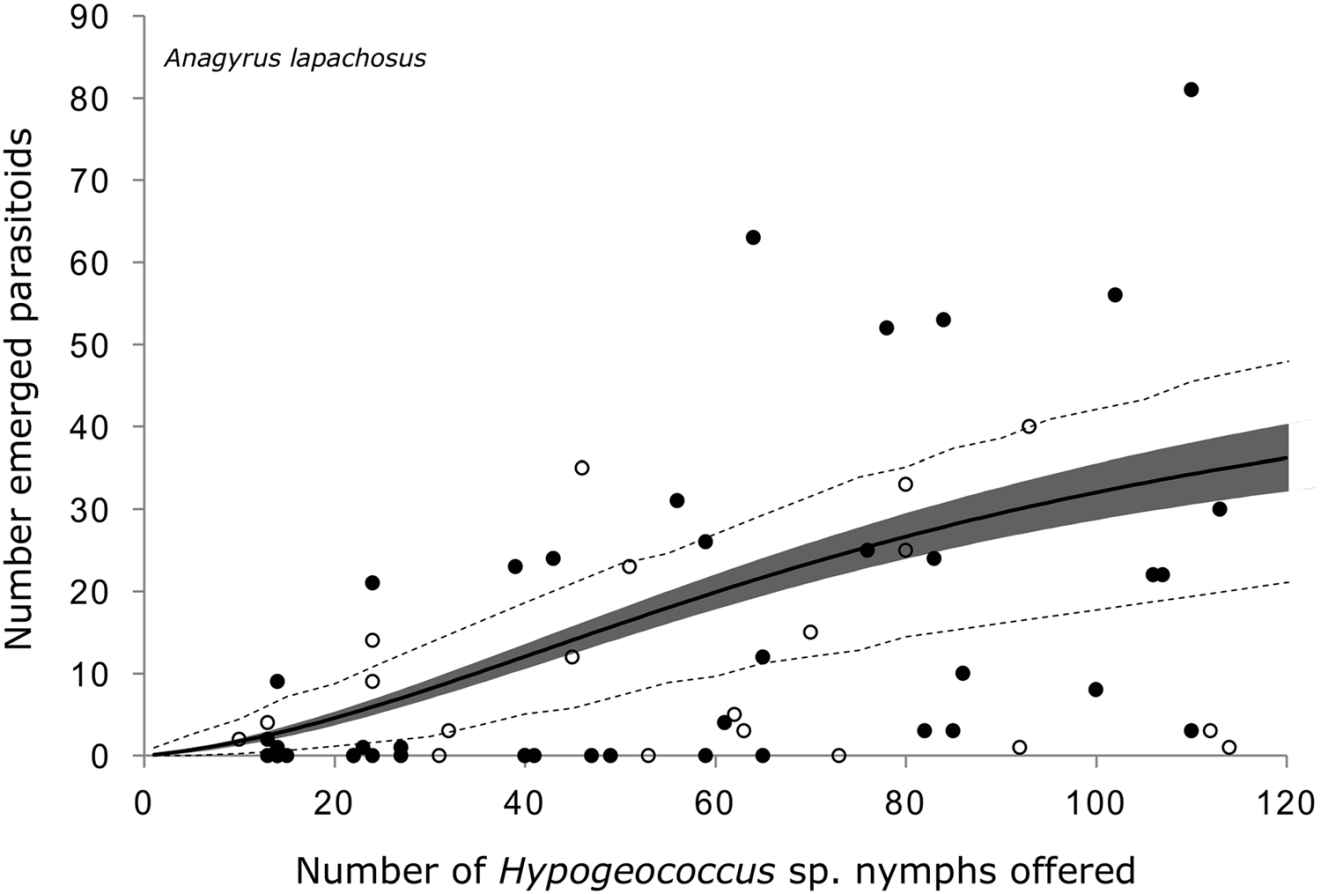
Observed functional response of the parasitoid *Anagyrus lapachosus* attacking *Hypogeococcus* sp. nymphs. Solid line indicates the mean estimation of functional response, grey area indicates its credibility interval, and dashed line indicates the a posteriori credibility interval for individual measurements. White circles are the observed number of emerged parasitoids in interaction experiments, while dark circles are the number of parasitoids emerged in experiments without interaction.

**Figure 3:**
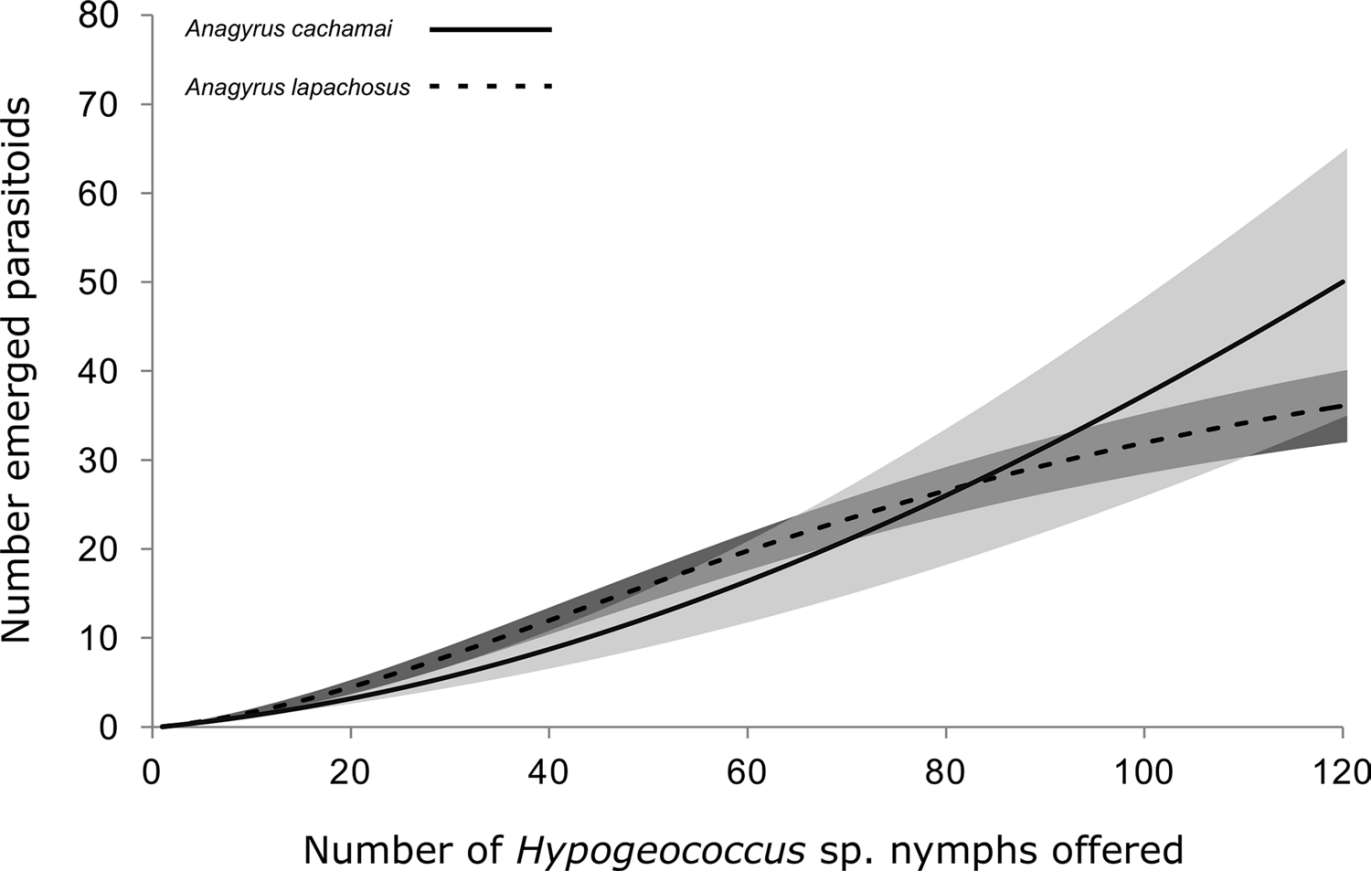
Observed functional response curves of two competing parasitoid species, *Anagyrus cachamai* and *A. lapachosus*, attacking *Hypogeococcus* sp. nymphs. The black line represents the average estimated functional response for *A. cachamai* and the dotted line for *A. lapachosus*. The light gray area indicates the credibility interval for the estimated functional response of *A. cachamai*, and the dark gray area shows the credibility interval for *A. lapachosus*.

When the components of the eight selected models were analyzed, we found that in terms of functional response, all the selected models had generalized type III functional response (GP), the models without host depletion (NHD) were selected in 66.7% of the iterations, and those GP that incorporated Roger’s host depletion correction (HD) were selected for the remaining 33.3% of iterations (Table 2). In the GP models (Supplementary, Equation (1.3)), the attack rate (*a*) follows a linear relationship to the density of the hosts offered (*p*). The initial attack rate of an inexperienced female (*b*) was 0.10 ± 0.03 d^−1^ for *A. cachamai* females and 0.11 ± 0.05 d^−1^ for *A. lapachosus* females, and no difference was observed between the species. The attack rate was higher for *A. lapachosus* than for *A. cachamai* females (0.007 ± 0.001 vs. 0.003 ± 0.001 d^−1^), while the handling time (*H*) was shorter for *A. cachamai* than *A. lapachosus* females (0.002 ± 0.002 vs. 0.019 ± 0.004 d) (Table 3). Therefore, the handling time of *A. cachamai* differed by an order of magnitude with respect to that found in *A. lapachosus*, while the attack rate, as a function of the number of nymphs offered, was in the same order of magnitude (Figs. 1 and 2). The estimated functional response curves indicated that *A. lapachosus* was the most efficient species at densities of *Hypogeococcus* sp. nymphs below 83, while above this value, *A. cachamai* was more efficient (Fig. 3).

**Table 3:** A posteriori mean ± standard deviation of the species-specific parameters of the selected models for two parasitoids, *Anagyrus cachamai* and *A. lapachosus*, attacking *Hypogeococcus* sp.

Model	Increase in host mortality	Rejection of non-suitable hosts	Functional response			Competition		
Species	Mortality caused by multiparasitism: *m*	Unsuitable hosts: *s*	Attack rate of an inexperienced female:* b* (d^−1^)	Attack rate: *a* (d^−1^)	Handling time: *H* (d)	Strength	First arrival term (d^−1^)	Multiparasitism index
*Anagyrus cachamai*	0.14 ± 0.07	0.15 ± 0.07	0.10 ± 0.03	0.003 ± 0.001	0.002 ± 0.002	0 ± 0	− 0.9 ± 0.7	0.3 ± 0.2
*Anagyrus lapachosus*			0.11 ± 0.05	0.007 ± 0.001	0.019 ± 0.004	0.2 ± 0.7	− 0.7 ± 0.9	0.8 ± 0.1

Parameter calculation was averaged for the eight selected models based on their optimal balance between explanatory power and complexity. Physical units of the calculated parameters: d days, parameters without units are dimensionless.

The interference competition behavior models revealed that *A. lapachosus* was the species that showed the greatest strength of the two species (Table 3). The models that considered the existence of competitive advantage by the order of arrival to the host and incorporated the females’ ability to discriminate between parasitized and unparasitized hosts were selected in 100% of the iterations (Table 2). The species arriving first to the host had a competitive disadvantage over the one that arrived second (Table 3). The competitive strength of the first arrival species was calculated as: µ_1_ + *h*_1_ (µ: strength and *h*: first arrival term). With this information, we calculated the proportion of times in which the strength of the second species arriving at the host was superior to the strength of the species that arrived first, and therefore won the competition according to Equation (3) (Supplementary). The competitive strength of *A. cachamai* females when they arrived first to *Hypogeococcus* sp. nymphs was − 0.9 ± 0.7 (µ_1_ = 0.0 + *h*_1_ = − 0.9; see Table 3). Since the competitive strength of *A. lapachosus* was 0.2 ± 0.7, there was a mean difference of strength of 1.1 between the two species, indicating that *A. lapachosus* was able to win a proportion of 0.9 (0.5–1) interactions against *A. cachamai* when arriving second. When the first arriving parasitoid was *A. lapachosus,* its competitive strength was − 0.5 ± 1.1 (µ_1_ = 0.2 + *h*_1_ = − 0.7; see Table 3). As the strength of *A. cachamai* was 0 ± 0 and the difference of strength was − 0.5, when *A. cachamai* arrived second to the host it was able to win 0.7 (0.3–0.9) of the contests. The behavior of avoidance/random/preference of the already parasitized hosts revealed that *A. cachamai* females avoided ovipositing in the already parasitized nymphs whereas *A. lapachosus* females preferred the parasitized hosts (Table 3).

The models that considered rejection by females of unsuitable hosts were selected in 44.4% of the iterations (Table 2). According to this result, a proportion of 0.15 of the nymphs offered to *A. cachamai* and *A. lapachosus* females were avoided by both parasitoid species. Models that considered increased mortality caused by multiparasitism were selected in 40.9% of the iterations (Table 2). This meant that some *Hypogeococcus* sp. nymphs died as a direct effect of *A. cachamai* or *A. lapachosus* female oviposition, regardless of parasitoid species. The increase in host mortality was 0.14 ± 0.07 in the first attack, raising this value with the number of attacks (*n*) as (1 − (0.14 ± 0.07))^*n*^ (according to Equation (8), Supplementary).

## Discussion

Our data provided evidence that *A. lapachosus* did practice intraguild predation on *A. cachamai*. The species differed in terms of their functional response, interference competitive strength, and host selection behavior. *Anagyrus cachamai* was the species that had the greatest ability to exploit the resource, while *A. lapachosus* was the strongest species in the interference competition. The functional response models highlighted a superior host exploitation ability for *A. cachamai*. On the other hand, the outcome of competition models indicated that asymmetric larval competition occurred between *A. cachamai* and *A. lapachosus*, with the latter outcompeting the former. Likewise, *A. lapachosus* females preferred parasitized mealybugs to unparasitized ones, while *A. cachamai* females avoided them. These behavioral differences played a key role in the wasp emergence patterns that were identified (Table 1).

The coexistence among species with different competitive strengths may be possible if the parasitoids possess differences in their competitive abilities^36,37^. In this context, it is expected that the weakest competitor presents the greatest ability to exploit the resource^38,39^. We found that *A. cachamai*, the less aggressive species, was the most efficient consumer since it had a shorter handling time. Although *A. lapachosus* females presented the highest change in the attack rate as a function of the number of nymphs offered, it was in the same order of magnitude as that observed in *A. cachamai*, while the difference observed in the handling time differed by an order of magnitude. Cusumano et al.^40^ found similar results for the interaction between *Trissolcus basalis* (Wollaston) (Hymenoptera: Scelionidae) and *Ooencyrtus telenomicida* (Vassiliev) (Hymenoptera: Encyrtidae), both egg parasitoids of *Nezara viridula* (L.) (Hemiptera: Pentatomidae). For this parasitoid-parasitoid interaction, *T. basalis* was the most efficient consumer, showing the shorter handling time, while *O. telenomicida* was better in larval competition. The authors suggested that *T. basalis*–*O. telenomicida* coexistence can be driven by a trade-off between host finding and competition.

Similar to many parasitoid species^41–43^, the order of arrival to the host affected the competitive strength of *A. cachamai* and *A. lapachosus*. *Anagyrus cachamai* females experienced a decrease in their competitive strength when the females arrived first to the host. The same pattern was observed in *A. lapachosus* females. However, the consequences for each parasitoid species were different. The second female species might have produced physical and chemical changes in the host’s environment to create conditions that favored its own larval survival in detriment to the parasitoid that arrived first^24,44^. For instance, the parasitoid female might inject viruses or toxins during oviposition^45^ or mechanically eliminate the immature competitor larva with its ovipositor^23^.

Regarding the behavior of attacking an already parasitized host, the strength of the second arriving species is expected to be higher than the one already inside the host^46^. Although the competitive strength of both *Anagyrus* species increased when they arrived second to the host, the proportion of times where *A. cachamai* arrived second to the host and won the competition against *A. lapachosus* was highly variable (0.7 (0.3–0.9)). *Anagyrus cachamai* females probably compensated their reduced competitive ability in the interference competition with a faster host manipulation and avoidance of parasitized hosts. As mentioned above, *A. lapachosus* females preferred parasitized nymphs to non-parasitized ones. The acceptance of hosts within the same trophic level is a mechanism to eliminate competitors, as well as a strategy to obtain high-protein or alternative hosts when the resource is scarce^2,17^. To our knowledge, our study is the first report of intraguild predation within the genus *Anagyrus* Howard. We hypothesized that *A. lapachosus* larvae behave either as predators of *A. cachamai* larvae during intrinsic competition, or, perhaps less likely, the former species could behave as a facultative hyperparasitoid. However, we have found no records of hyperparasitoid species in the genus *Anagyrus*, which is comprised entirely of primary parasitoids of various mealybugs*.*

Some of the selected models (44.4%) indicated that *A. cachamai* and *A. lapachosus* females did not attack a certain proportion of the hosts offered. Possibly, the hosts were not suitable for the development of their offspring. An alternative explanation may be that only 24–48 h old females were used, not considering the synovigenic behavior of the two parasitoid species^28^, where their egg load changed throughout the female’s lifespan. Transient egg limitation can make eggs more valuable than if the wasps never experienced a limitation, influencing females to be less likely to lay eggs in unsuitable hosts. Similarly, egg reabsorption often confers greater fitness than ovipositing in unsuitable hosts. To achieve a better understanding of the process or processes that affected host selection behavior of both *Anagyrus* species, it will be necessary to expand the interaction experiment design to include additional factors, for example, females of different ages.

Parasitoid impact on host population is underestimated when host mortality is not taken into account^47^. Both larval and adult parasitoids can induce host death following oviposition. When neither parasitoid nor host emerges, Abram et al.^47^ called it *non-reproductive effect*. However, host mortality cannot be easily measured, especially if it is not possible to detect the parasitoid via dissection or if the oviposition was interrupted and no egg was laid^48^. Currently, in biological control programs, the population consequences of non-reproductive mortality of hosts induced by their parasitoids and its effects in multiple-hosts systems are unknown^48,49^. Abram et al.^47^ proposed including the contribution of non-reproductive mortality both in models of host-parasitoid population dynamics, as in those that include multi-trophic interactions, in order to have a better understanding regarding its effect on the community interactions. With the methodological approach proposed in this study, we found that multi parasitism increased the death probability of the *Hypogeococcus* sp. nymphs. This result was also reported for other parasitoid species^50,51^. Host mortality increase can be caused by the physical damage produced by the increase in the number of stings with or without oviposited larvae per host, changes in its internal environment^52^, host rejection^48^, death of the egg or larva of the parasitoids that do not develop but end up killing the host^53^, and parasitized hosts being more susceptible to infections^54^.

Finally, to analyze the type of interactions that existed between *A. cachamai* and *A. lapachosus*, we used models both with host depletion (exponential decaying host density with consumption, according to Rogers^55^) or without it (the basic Holling’s models^56^). In our simulations, we found that one-third of the time, the Rogers models were selected, and another two-thirds, the Hollings were selected. These results suggest that indeed the host depletion is affecting the performance of the parasitoids, in an intermediate form between that proposed by Rogers and the original from Holling.

**Figure 4:**
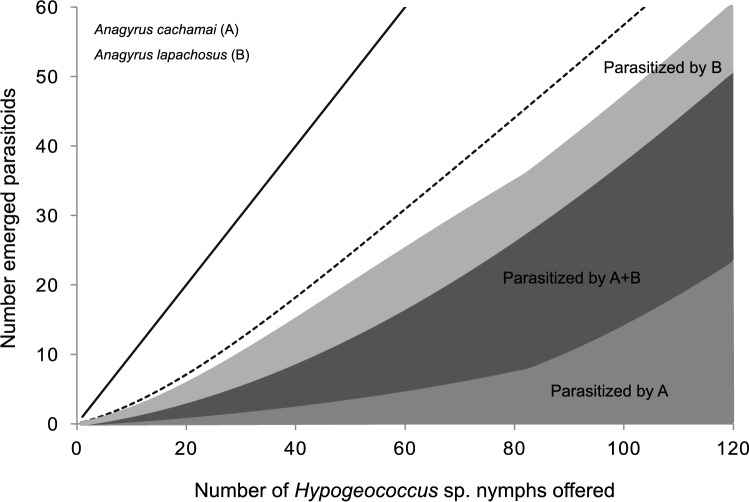
Expected functional response in the interaction between the parasitoids *Anagyrus cachamai* (**A**) and *A. lapachosus* (**B**), when the nymphs of *Hypogeococcus* sp. were first exposed to *A. cachamai*. Black line: number of nymphs offered is equal to the number of nymphs attacked by parasitoids. Dotted line: females of *A. lapachosus* do not have a host selection behavior; therefore, the number of nymphs attacked is at random.

**Figure 5:**
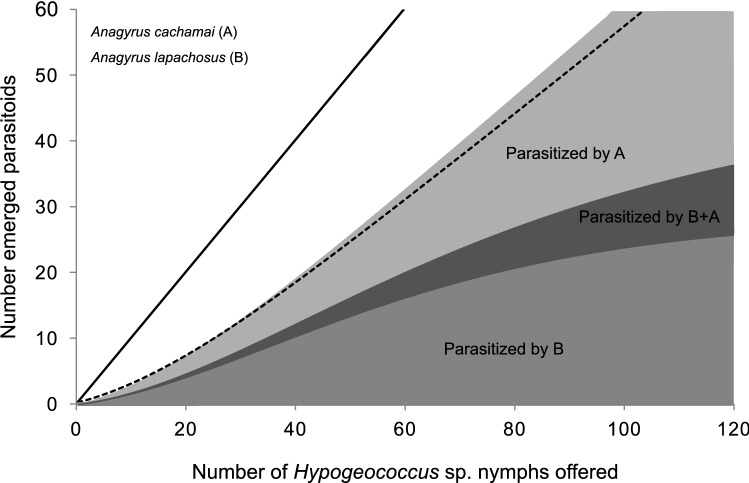
Expected functional response in the interaction between the parasitoids *Anagyrus cachamai* (**A**) and *A. lapachosus* (**B**), when the nymphs of *Hypogeococcus* sp. were first exposed to *A. lapachosus*. Black line: number of nymphs offered is equal to the number of nymphs attacked by parasitoids. Dotted line: females of *A. cachamai* do not have a host selection behavior; therefore, the number of nymphs attacked is at random.

The role that intraguild predation played on the interaction between *A. cachamai* and *A. lapachosus* and its consequences for the control of *Hypogeococcus* sp. revealed two possible scenarios that depended on the order in which the *Hypogeococcus* sp. nymphs were exposed to these parasitoids. If the nymphs were first exposed to *A. cachamai* and then to *A. lapachosus*, given that *A. lapachosus* females preferred the parasitized nymphs, the degree of overlap between these two species would be high. As a consequence, control by the parasitoids would be lower than expected when the interaction is at random, as it is in the case when *A. lapachosus* females do not have a host selection behavior (Fig. 4). If the order of exposure of nymphs to parasitoids was reversed, the overlap between *A. cachamai* and *A. lapachosus* would decrease. The females of *A. cachamai* had a greater ability to exploit the resource than those of *A. lapachosus*, and the former species avoided the already parasitized nymphs, the total number of parasitized nymphs would increase, exceeding that expected by random (Fig. 5).

In conclusion, our models predicted that a multiple species release strategy would likely produce more control of the pest host than a single species release when *A. lapachosus* was released first. To obtain a more comprehensive knowledge of the interactions between these two parasitoids on the suppression of *Hypogeococcus* sp., investigations on continuous generations should be conducted. It is also necessary to identify and characterize the natural enemies present in the release area given that negative interactions with other parasitoids and/or predators could adversely affect pest control. Our next goal is to investigate the interactions between *A. cachamai* and *A. lapachosus* on continuous generations in field manipulative experiments.

## Supplementary Information

Supplementary Information.
