# Supplementary material for: Influence of competition and intraguild predation between two candidate biocontrol parasitoids on their potential impact against Harrisia cactus mealybug, Hypogeococcus sp. (Hemiptera: Pseudococcidae)
Source: Sci Rep. 2021 Jun 28;11:13377. doi: 10.1038/s41598-021-92565-6 (PMC8239034; doi:10.1038/s41598-021-92565-6)
Supplement: Supplementary file 1 — Supplementary Information. [file 41598_2021_92565_MOESM1_ESM.docx]

**Supplementary Information**

**Article**: Influence of competition and intraguild predation between two candidate biocontrol parasitoids on their potential impact against Harrisia cactus mealybug, *Hypogeococcus* sp. (Hemiptera: Pseudococcidae)

**Authors:** María B. Aguirre^1,2,*^, Octavio A. Bruzzone^2,3^, Serguei V. Triapitsyn^4^, Hilda Diaz-Soltero^5^, Stephen D. Hight^6^, and Guillermo A. Logarzo^1^

**Affiliation and addresses of the authors:**

^1^Fundación para el Estudio de Especies Invasivas (FuEDEI), Hurlingham, Buenos Aires, Argentina.

^2^Consejo Nacional de Investigaciones Científicas y Técnicas, Ciudad Autónoma de Buenos Aires, Argentina.

^3^Instituto Nacional de Tecnología Agropecuaria (INTA), Estación Experimental Agropecuaria Bariloche, San Carlos de Bariloche, Río Negro, Argentina.

^4^Department of Entomology, University of California, Riverside, California, 92521, USA.

^5^Animal and Plant Health Inspection Service USDA, San Juan, Puerto Rico.

^6^USDA-ARS-CMAVE at Center for Biological Control, Florida A&M University, Tallahassee, Florida, 32308, USA.

^*^Corresponding author: María B. Aguirre, redbell_@hotmail.com

**Appendix**

Description of models used to analyze the interaction between the parasitoids *A. cachamai* and *A. lapachosus*.

Functional response models

We proposed three levels of functional response, depending on the number of parameters of the model. We considered the type I, type II, and type III generalized functional responses, with and without correction per host depletion. The process of identifying the type of functional response was identified in terms of complexity of the model (in the number of parameters), instead of the shape of the curve. The proposed type III functional response models may have resulted in a range of different curve shapes, beyond the sigmoidal curve of the Holling^1^ model.

*Models without host depletion* (NHD)

1.1 Linear type I functional response ^1^. The parasitoids attack a constant proportion of the offered hosts, with no upper limit in the range of offered hosts during the experiment:

$$R(p)=apt (1.1)$$

where *p* is the number of hosts offered, *a* is the attack rate, and *t* is the time the hosts are exposed to a parasitoid species (1 day).

1.2 Hyperbolic Holling’s type II functional response. The number of consumed hosts depends also on a second parameter, called handling time (*H*):

$$R(p)=apt / 1+aHp (1.2)$$

1.3 Order two polynomial generalized type III functional response (GP). We propose a generalization of the quadratic type III functional response of Holling ^1^, in which the attack rate, or efficiency, changes linearly with the number of hosts. The functional response equation was defined as:

$$R(p)=(apt+b) p / 1+H (ap+b) p (1.3)$$

with $0<ap+b\leq1$

where *a* (the slope) is the female attack rate which changes as a function of the number of nymphs offered (*p*), *b* is the attack rate when a female emerges with *p* = 0. The main difference with Holling’s functional response is the intercept term (the efficiency at zero hosts (*b*), which is assumed to be equal to 0). In this model, depending on the values that the slope *a* takes, a hyperbolic curve would be produced similar to the type II curve with *a ~* 0*,* or a sigmoidal curve with *a* ~ 1. With *b =* 0 and *a =* 1, the model reduces to the Hollings type III.

1.4 Generalized type III functional response (G) ^2^. This model is another generalization of the functional response curve, but now, the third parameter is the exponent, leading to an equation that can be written as:

$$R(p)=(tap^{s}) / 1+Hap^{s} (1.4)$$

where *s* = 1 + *q, q* representing the attack component that converts hyperbolic type II functional response (*q* = 0) into sigmoid type III functional response (*q* ˃ 0). With the exponent lower or near 1, it results in the Hollings type II functional response, with exponents higher than 1, a sigmoidal curve. With the exponent equal 2, the model reduces to the classical Holling’s type III curve.

*Models with host depletion* (HD)

For the Hyperbolic Holling’s type II functional response model, and both type III generalized functional response (GP and G) models, a version with host depletion was also proposed, according to the random predator equation of Rogers ^3^. Rogers’ models correct the decrease in host density produced by the consumption by the parasitoid, so we proposed it in the case that host depletion could affect the estimation of the functional response parameters.

Competition models

For interspecific parasitoid interaction experiments between species 1 and 2, the hosts were classified into four categories: attacked only by species 1, attacked only by species 2, attacked by both species, and not attacked. The expected number of individuals emerged on each experiment as a function of hosts (*p*) available was analyzed using equation (1):

$E_{1/2}\left( p \right)=R_{10}(p)+R_{12}(p)\left( 1-w_{12} \right)$

The proportion of times in which species 1 won in the competition against species 2 was calculated according to Thurstone’s model case V ^4^ using the following equation:

$$w_{12}={P(S}_{1}>S_{2}) (2)$$

where *S*_1_ and *S*_2_ are the strength in terms of competitive power of species 1 and 2 respectively. *P* (*S*_1_ > *S*_2_*)* is the proportion of times in which the strength of 1 is greater than the strength of 2 (and therefore wins the competition by interference). Given that the strength is not a constant number, but a normally distributed random variable, thus *P* (*S*_1_ > *S*_2_) is the difference of two Gaussian distributions, so $P(S_{1}>S_{2})\sim N(\mu_{12}, 1).$ If a constant value is added to the first arriving species in order to estimate the decrease or increase in parasitoid competitive strength, the following equation is obtained:

$P(S_{1}>S_{2})\sim N({h_{1}+\mu}_{1}-\mu_{2}, 1) (3)$

where $\mu_{1}-\mu_{2}$ is the mean competitive strength difference between species 1 and 2*,* and *h*_1_ is the first arrival term for the first arriving parasitoid (here species 1). Positive values of *h_1_* means advantage for the first arriving parasitoid, and negative values means an advantage for the second arriving species. In Thurstone’s case V model, $\sigma_{1}=\sigma_{2}$ was set to simplify the equation, and we assumed that $\sigma_{1}^{2}= \sigma_{2}^{2}= \sqrt{2/2}, so that \sigma_{12}^{2}=1.$

*Parasitoid superposition models*

Some parasitoids have the ability to discriminate between parasitized and unparasitized hosts ^5,6^, thereby deciding whether to attack parasitized hosts or not. In cases where multiparasitism succeeds, it becomes a viable strategy ^7^. Therefore, we proposed a new host selection model in which we considered different host-selection behaviors:

- *Random superposition:* The number of hosts that were attacked by both species, and the number of hosts in which the competition occurs is assumed to be the product of random superposition and estimated as the product of the proportion of hosts parasitized by species 1 and 2, multiplied by the total number of hosts offered:

$R_{1}(p)\cap R_{2}(p)=R_{1}(p)R_{2}(p) (4)$

- *Avoidance superposition*: If the second parasitoid avoids already parasitized hosts, the parasitoid first uses the available “free” hosts, and only after they are completely depleted, the female uses the hosts parasitized by the previous species. Equation (4) was modified as:

$$R_{1}\left( p \right){\cap R}_{2}\left( p \right)=\left\{ \begin{aligned} {(R}_{1}{\left( p \right)+R}_{2}{(p))-p, if (R}_{1}{\left( p \right)+R}_{2}(p))>p \\ 0 , otherwise \end{aligned} (5) \right.$$

- *Preference superposition:* If the second parasitoid prefers the parasitized hosts, the female uses these hosts first, and after they are completely depleted, it switches to the “free” hosts. Now the number of hosts attacked by both species follows the equation:

$$R_{1}\left( p \right)\cap R_{2}\left( p \right)=\left\{ \begin{aligned} R_{2}\left( p \right), ifR_{1}\left( p \right)>R_{2}\left( p \right) \\ R_{1}\left( p \right),ifR_{1}\left( p \right)<R_{2}\left( p \right) \end{aligned} \right. \left( 6 \right)$$

Since the behavior of avoidance/random/preference is species-specific, the best way to integrate the three models into a single one was to create an index of multiparasitism. This index is similar to the correlation coefficient, with a range from 0 to 1; where 0 means complete avoidance (the number of hosts attacked by both species is minimal), 0.5 means random (without host-selection behavior), and 1 means complete preference for parasitized hosts (the number of hosts attacked by both species is maximum). As the index can take intermediate values, the proportion of preference or avoidance vs. random can be averaged. Thus, the final equation describing the number of hosts attacked was developed by putting together equations (4), (5) and (6) as follows:

$$R_{1}\left( p \right)\cap R_{2}\left( p \right)=\left\{ \begin{aligned} N_{r}\left( 1-2i \right)+N_{s}2i, if i<0.5 \\ N_{r}\left( 1-2i \right)+N_{a}2i, if i>0.5 \\ N_{r} , otherwise \end{aligned} (7) \right.$$

where *i* is the multiparasitism index which ranges from 0 to 1, *N_r_* is the expected number of hosts attacked by both species under a completely random assumption, as in equation (4), *N_s_* is the same variable under complete superposition assumption as in equation (6), and *N_a_* is the same variable but now under complete avoidance assumption as in equation (5).

*Models with rejection of non-suitable hosts and increase in host mortality*.

To account for the existence of unsuitable hosts, and for the increase in host mortality caused by multiparasitism, two additional terms were added to equation (1) to form the following:

$${Ec}_{1/2}\left( p \right)=E_{1/2}\left( p \right)\left( 1-s \right)\left( 1-m \right)^{n} (8)$$

where *Ec* is the expected number of emerged parasitoids (*E*) after correcting by mortality and unsuitability of hosts, *s* is the proportion of hosts considered unsuitable for the parasitoids, *m* is the proportion of hosts that died as a consequence of parasitism, and *n* is the number of parasitoids to which a certain group of hosts were exposed.

The final model can be described as:

$${Ec}_{1/2}\left( p \right)=UM (R+\left( IW \right)) (9)$$

where the terms of the model are described as follows: *U* is the proportion of unsuitable hosts term from equation (8), *M* is the increased mortality caused by parasitism, also from equation (8), *R* is the functional response model which can be either type I, II, III (GP and G), with host depletion (HD) or without host depletion (NHD) (see functional response models section), *I* is the parasitoid superposition model from equation (7), and *W* is the Thurstone competition term without competitive advantage by the order of arrival to the host from equation (2) or with competitive advantage, *W_A_*, from equation (3). Different equations for these terms (*U*, *M*, *R*, *I*, *W*) compose the model selection procedure, as in the Table S1.

**Table S1.** Models used to analyze the interactions (competition and intraguild predation) between two species of *Hypogeococcus* sp. parasitoids.

| **Models of functional response** |  | **Models with increase in host mortality** |  | **Models of competition** | | | | | | | | | |
| --- | --- | --- | --- | --- | --- | --- | --- | --- | --- | --- | --- | --- | --- |
|  |  |  |  | Absent | Present, without parasitoids superposition | Present, without parasitoids superposition and competitive advantage by the order of arrival to the host | Present, with parasitoids superposition and competitive advantage by the order of arrival to the host |  | Absent | Present, without parasitoids superposition | Present, without parasitoids superposition and competitive advantage by the order of arrival to the host | Present, with parasitoids superposition and competitive advantage by the order of arrival to the host |  |
|  |  |  |  | **Models without rejection of non-suitable hosts** | | | |  | **Models with rejection of non-suitable hosts** | | | |  |
| FRI, NHD |  | Absent |  | *E_c1/2_ (p)=R_I_* | *E_c1/2_ (p)=R_I_+W* | *E_c1/2_ (p)=R_I_+W_A_* | *E_c1/2_ (p)=R_I_+IW_A_* |  | *E_c1/2_ (p)=U (R_I_)* | *E_c1/2_ (p)=U (R_I_+W)* | *E_c1/2_ (p)=U (R_I_+W_A_)* | *E_c1/2_ (p)=U (R_I_+IW_A_)* |  |
| FRII, NHD |  |  |  | *E_c1/2_ (p)=R_II_* | *E_c1/2_ (p)=R_II_+W* | *E_c1/2_ (p)=R_II_+W_A_* | *E_c1/2_ (p)=R_II_+IW_A_* |  | *E_c1/2_ (p)=U (R_II_)* | *E_c1/2_ (p)=U (R_II_+W)* | *E_c1/2_ (p)=U (R_II_+W_A_)* | *E_c1/2_ (p)=U (R_II_+IW_A_)* |  |
| FRII, HD |  |  |  | *E_c1/2_ (p)=R_II_* | *E_c1/2_ (p)=R_II_+W* | *E_c1/2_ (p)=R_II_+W_A_* | *E_c1/2_ (p)=R_II_+IW_A_* |  | *E_c1/2_ (p)=U (R_II_)* | *E_c1/2_ (p)=U (R_II_+W)* | *E_c1/2_ (p)=U (R_II_+W_A_)* | *E_c1/2_ (p)=U (R_II_+IW_A_)* |  |
| FRIII GP, NHD |  |  |  | *E_c1/2_ (p)=R_III_* | *E_c1/2_ (p)=R_III_+W* | *E_c1/2_ (p)=R_III_+W_A_* | *E_c1/2_ (p)=R_III_+IW_A_* |  | *E_c1/2_ (p)=U (R_III_)* | *E_c1/2_ (p)=U (R_III_+W)* | *E_c1/2_ (p)=U (R_III_+W_A_)* | *E_c1/2_ (p)=U (R_III_+IW_A_)* |  |
| FRIII GP, HD |  |  |  | *E_c1/2_ (p)= R_III_* | *E_c1/2_ (p)=R_III_+W* | *E_c1/2_ (p)=R_III_+W_A_* | *E_c1/2_ (p)=R_III_+IW_A_* |  | *E_c1/2_ (p)=U (R_III_)* | *E_c1/2_ (p)=U (R_III_+W)* | *E_c1/2_ (p)=U (R_III_+W_A_)* | *E_c1/2_ (p)=U (R_III_+IW_A_)* |  |
| FRIII G, NHD |  |  |  | *E_c1/2_ (p)= R_III_* | *E_c1/2_ (p)=R_III_+W* | *E_c1/2_ (p)=R_III_+W_A_* | *E_c1/2_ (p)=R_III_+IW_A_* |  | *E_c1/2_ (p)=U (R_III_)* | *E_c1/2_ (p)=U (R_III_+W)* | *E_c1/2_ (p)=U (R_III_+W_A_)* | *E_c1/2_ (p)=U (R_III_+IW_A_)* |  |
| FRIII G, HD |  |  |  | *E_c1/2_ (p)= R_III_* | *E_c1/2_ (p)=R_III_+W* | *E_c1/2_ (p)=R_III_+W_A_* | *E_c1/2_ (p)=R_III_+IW_A_* |  | *E_c1/2_ (p)=U (R_III_)* | *E_c1/2_ (p)=U (R_III_+W)* | *E_c1/2_ (p)=U (R_III_+W_A_)* | *E_c1/2_ (p)=U (R_III_+IW_A_)* |  |
|  |  |  |  |  |  |  |  |  |  |  |  |  |  |
| FRI, NHD |  | Present |  | *E_c1/2_ (p)=M (R_I_)* | *E_c1/2_ (p)=M (R_I_+W)* | *E_c1/2_ (p)=M (R_I_+W_A_)* | *E_c1/2_ (p)=M (R_I_+IW_A_)* |  | *E_c1/2_ (p)=UM (R_I_)* | *E_c1/2_ (p)=UM (R_I_+W)* | *E_c1/2_ (p)=UM (R_I_+W_A_)* | *E_c1/2_ (p)=UM (R_I_+IW_A_)* |  |
| FRII, NHD |  |  |  | *E_c1/2_ (p)=M (R_II_)* | *E_c1/2_ (p)=M (R_II_+W)* | *E_c1/2_ (p)=M (R_II_+W_A_)* | *E_c1/2_ (p)=M (R_II_+IW_A_)* |  | *E_c1/2_ (p)=UM (R_II_)* | *E_c1/2_ (p)=UM (R_II_+W)* | *E_c1/2_ (p)=UM (R_II_+W_A_)* | *E_c1/2_ (p)=UM (R_II_+IW_A_)* |  |
| FRII, HD |  |  |  | *E_c1/2_ (p)=M (R_II_)* | *E_c1/2_ (p)=M (R_II_+W)* | *E_c1/2_ (p)=M (R_II_+W_A_)* | *E_c1/2_ (p)=M (R_II_+IW_A_)* |  | *E_c1/2_ (p)=UM (R_II_)* | *E_c1/2_ (p)=UM (R_II_+W)* | *E_c1/2_ (p)=UM (R_II_+W_A_)* | *E_c1/2_ (p)=UM (R_II_+IW_A_)* |  |
| FRIII GP, NHD |  |  |  | *E_c1/2_ (p)=M (R_III_)* | *E_c1/2_ (p)=M (R_III_+W)* | *E_c1/2_ (p)=M (R_III_+W_A_)* | *E_c1/2_ (p)=M (R_III_+IW_A_)* |  | *E_c1/2_ (p)=UM (R_III_)* | *E_c1/2_ (p)=UM (R_III_+W)* | *E_c1/2_ (p)=UM (R_III_+W_A_)* | *E_c1/2_ (p)=UM (R_III_+IW_A_)* |  |
| FRIII GP, HD |  |  |  | *E_c1/2_ (p)=M (R_III_)* | *E_c1/2_ (p)=M (R_III_+W)* | *E_c1/2_ (p)=M (R_III_+W_A_)* | *E_c1/2_ (p)=M (R_III_+IW_A_)* |  | *E_c1/2_ (p)=UM (R_III_)* | *E_c1/2_ (p)=UM (R_III_+W)* | *E_c1/2_ (p)=UM (R_III_+W_A_)* | *E_c1/2_ (p)=UM (R_III_+IW_A_)* |  |
| FRIII G, NHD |  |  |  | *E_c1/2_ (p)=M (R_III_)* | *E_c1/2_ (p)=M (R_III_+W)* | *E_c1/2_ (p)=M (R_III_+W_A_)* | *E_c1/2_ (p)=M (R_III_+IW_A_)* |  | *E_c1/2_ (p)=UM (R_III_)* | *E_c1/2_ (p)=UM (R_III_+W)* | *E_c1/2_ (p)=UM (R_III_+W_A_)* | *E_c1/2_ (p)=UM (R_III_+IW_A_)* |  |
| FRIII G, HD |  |  |  | *E_c1/2_ (p)=M (R_III_)* | *E_c1/2_ (p)=M (R_III_+W)* | *E_c1/2_ (p)=M (R_III_+W_A_)* | *E_c1/2_ (p)=M (R_III_+IW_A_)* |  | *E_c1/2_ (p)=UM (R_III_)* | *E_c1/2_ (p)=UM (R_III_+W)* | *E_c1/2_ (p)=UM (R_III_+W_A_)* | *E_c1/2_ (p)=UM (R_III_+IW_A_)* |  |

*U* is the proportion of unsuitable hosts term, *M* is the increased mortality caused by parasitism, *R* is the functional response model which can be either type I, II, III (GP and G), with host depletion (HD) or without host depletion (NHD), *I* is the parasitoid superposition model, and *W* is the Thurstone competition term without competitive advantage by the order of arrival to the host or with competitive advantage, *W_A_*. Different equations for these terms (*U*, *M*, *R*, *I*, *W, W_A_*) compose the model selection procedure.

**References**

1. Holling, C. S. Some characteristics of simple types of predation and parasitism. *Can. Entomol.* **91**, 385–398. https://doi.org/10.4039/Ent91385-7 (1959).

2. Kalinkat, G. *et al.* Body masses, functional responses and predator–prey stability. *Ecol. Lett.* **16**, 1126–1134. <https://doi.org/https://doi.org/10.1111/ele.12147> (2013).

3. Rogers, D. Random search and insect population models. *J. Anim. Ecol.* **41**, 369–383. <https://doi.org/10.2307/3474> (1972).

4. Thurstone, L. L. A law of comparative judgment. *Psychol. Rev.* **34**, 273 (1927).

5. van Lenteren, J. Host discrimination by insect parasitoids. In *Semiochemicals. Their Role in Pest Control* (eds Nord, D. A., Jones, R. & Lewis, W. J.)*,* 153–179 (1981).

6. Wylie, H. G. Discrimination between parasitized and unparasitized house fly pupae by females of *Nasonia vitripennis* (Walk.) (Hymenoptera: Pteromalidae). *Can. Entomol.* **97**, 279–286. https://doi.org/10.4039/Ent97279-3 (1965).

7. Hamelin, F., Bernhard, P. & Wajnberg, É. Superparasitism as a differential game. *Theor. Popul. Biol.* **72**, 366–378. https://doi.org/https://doi.org/10.1016/j.tpb.2007.07.005 (2007).
